# PEDOT/PSS-Halloysite Nanotubes (HNTs) Hybrid Films: Insulating HNTs Enhance Conductivity of the PEDOT/PSS Films

**DOI:** 10.1038/srep18641

**Published:** 2015-12-21

**Authors:** Hu Yan, Ping Zhang, Juan Li, Xiao-Li Zhao, Ke Zhang, Bing Zhang

**Affiliations:** 1 College of Chemistry and Molecular Engineering, Zhengzhou University, 100 Kexue Road, Zhengzhou, China; 2 School of Chemical Engineering and Energy, Zhengzhou University, 100 Kexue Road, Zhengzhou, China

## Abstract

We have for the first time found that completely insulating Halloysite nanotubes (HNTs) significantly enhance electrical conductivity of PEDOT/PSS films by simply mixing. Based on this accident finding we have created highly porous and conductive PEDOT/PSS films hybridized with the HNTs. Through further optimization of the mixing condition we have obtained flexible and conductive hybrid films with high specific surface area. Based on experimental evidences we proposed a plausible mechanism of the phenomenon where the PEDOT/PSS colloidal particle with particle size of several tens nanometers well pack at the nano-channels into well-ordered structures of PEDOT/PSS particles, which show conductivity as higher as several order of magnitude than that of PEDOT/PSS particles in outside of the HNTs.

Poly(3,4-ethylenedioxythiophene)/poly(4-styrenesul-fonate) (PEDOT/PSS), one of the most commercially successful conducting polymers, has attracted wide attentions from both academic and technological aspects since the excellent conductivity, transparency and thermal stability coupled with the merit of low cost wet-process^1^,^2^,^3^,^4^,^5^,^6^,^7^. On the one hand, it is well known that addition of certain organic solvent such as dimethyl sulfoxide (DMSO) and ethylene glycol (EG) during preparation of the PEDOT/PSS films enables to significantly improve conductivity of the PEDOT/PSS films^8^,^9^,^10^,^11^,^12^,^13^. Post-treatments of the PEDOT/PSS films by certain organic compounds such as formic acid (FA), sulfuric acid and propanol also significantly improve the conductivity of the PEDOT/PSS films^14^. Hybridization with carbon nanotubes also is a common strategy to improve both conductivity and mechanical properties of the PEDOT/PSS thin films^15^.

On the other hand, Halloysite nanotubes (HNTs) have also attracted much attention due to their molecule-storage ability, excellent mechanical strength and thermal stability. HNTs consist of one alumina octahedron sheet and one silica tetrahedron sheet in 1:1 stoichiometric ratio^16^,^17^,^18^,^19^,^20^,^21^,^22^, both sheets bending as double layers into hollow nanotubular structure in the submicrometer range and large specific surface area^16^. It also known that clay minerals are used as pillars to improve mechanical properties of polymers^20^. To improve mechanical strength of the PEDOT/PSS films we investigated hybridization of the PEDOT/PSS with the HNTs. In this investigation we have accidently but for the first time found that conductivity of the PEDOT/PSS films are significantly improved by addition of the insulating HNTs. Herein we report systematic investigations on the PEDOT/PSS-HNTs hybrid films in terms of electrical conductivity and mechanism of conductivity enhancement. On basis of transmittance electron microscopic (TEM) data and plot of conductivity against content of NHTs in the hybrid films as well as specific surface area measured by BET adsorption we propose a novel plausible mechanism of the conductivity enhancement of the PEDOT/PSS films by the HNTs-addition.

Certain amount of HNTs was dispersed in the PEDOT/PSS dispersion (Clevios PH1000) and stirred for 24 h at room temperature. The HNTs-mixed PEDOT/PSS dispersion was drop-casted on poly(ethylene terephthalate) (PET) sheets. The PEDOT/PSS-HNTs hybrid films on the PET sheets were first dried at 50 °C for 1 h, and then at 120 °C for 15 min. The ethylene glycol (EG)-treated films were prepared by using 7% EG-added PEDOT/PSS dispersion. Other procedures are the same with the above-mentioned method. The formic acid (FA)-treated films were prepared as follows: The PEDOT/PSS-HNTs hybridized films were immersed in FA for 10 min, and then dried at 140 °C for 5 min. To improve the flexibility of the hybrid films certain amount of polyethylene glycol (PEG) was added to the dispersion and the films were prepared in the same manner. The films were further treated with the FA. The experimental procedures were described in Supporting Information (SI) in detail.

The electrical conductivity of the films was measured by a standard four-probe method with a Resist Meter (Lorester (MCP-T610). The X-ray diffraction (XRD, Bruker AXS, D8 ADVANCE) was measured to characterize the crystalline structure. The size and the morphology of the PEDOT/PSS-HNTs films were determined by a transmission electron microscope (TEM, FEITECNAIG2) and a scanning electron microscope (SEM, JEM-2100cx). BET isothermal curves were measured with a High Speed Automated Surface Area and Pore Size Analyzer (BET, Nova 2000e) by using nitrogen gas as an adsorbent. UV-Vis-near IR spectra of the hybrid films were measured by reflectance mode with a UV-Vis-near IR spectroscope (Agilent AU12320005). The detailed measurements including other analyses also were described in SI. FT-IR spectra of the pure HNTs and the hybrid films were measured with a FT-IR spectrometer (Nicolet, NEXUS-470) by using pellet samples compressed with KBr.

Preparation of the PEDOT/PSS-HNTs hybrid film by varying content of HNTs and EG was optimized in terms of conductivity, specific surface area and flexibility of the film, which will be discussed in detail. Figure 1 shows SEM image of the PEDOT/PSS-HNTs hybrid film prepared using PEDOT/PSS dispersion after adding 4% HNTs and 7% EG. The HNTs had “stick”-like shape in the SEM (see Fig. S3B), while the hybrid film had “stick-blended cake”-like one in the lower magnified SEM. However, the film also mainly had “stick”-like one at a close look (inset in Fig. 1). Based on a calculation from composition of the PEDOT/PSS dispersion the HNTs was 75.5% in the hybrid film, which is agreement with the SEM observation (inset). Although the high content of insulating HNTs the hybrid film had a moderately high conductivity, i.e., 47 S/cm with a high specific surface area (89 m^2^/g) and a moderately good flexibility. XRD patterns of the pure HNTs and the hybrid film revealed that the hybrid film formed by physical mixing of HNTs and PEDOT/PSS, as shown in Fig. 2 (also see Fig. S5).

The hybrid film was optimized in terms of conductivity and surface area, changing the content of HNTs in the film but without EG. Figure 3 shows plots of electrical conductivity of the PEDOT/PSS-HNTs hybrid films against content of HNTs in the films, whose experiment was repeated for five times. Surprisingly but interestingly, it is found that the conductivity of the film increased from 0.2 to 0.6 S/cm with increasing the content of HNTs from zero to 75.5%. It is noteworthy that HNTs is electrically insulating, therefore, then commonly believed that the conductivity of the hybrid film should be decreased with increasing the content of the insulating HNTs on volume percentage-basis when mixing insulating materials into conducting polymer films. It is considered that such enhancement of conductivity occurs through a novel mechanism which should be different from those of organic solvent-addition/-treatment^6^,^9^ or carbon nanotube-addition^14^. Detailed mechanism will be discussed later. When the content of HNTs further increased from 75.5 to 79.4% the conductivity of the hybrid films decreased from 0.6 to 0.2 S/cm, indicating common phenomenon when mixing insulating component into conductive matrix. Firstly, we have carefully characterized PEDOT/PSS-HNTs hybrid film with 75.5% HNTs by various analytic tools.

The BET surface area of the HNTs was 104 m^2^/g while the PEDOT/PSS-HNTs hybrid film was 64 m^2^/g (see Fig. S6). It is considered that the reduction of the surface area is due to both filling PEDOT/PSS in the channels of the NHTs and less content of the NHTs in the sample. It will be discussed later in the mechanism of conductivity enhancement. The hybrid films were treated with FA or added EG during preparation of the films. Both treatments significantly enhanced the conductivity of the hybrid films (see Fig. S3C,D). Highly flexible films were prepared by adding poly(ethylene glycol) (PEG) in the NHTs-contained PEDOT/PSS dispersion. As-prepared films were easily peeled from the substrate by immersing in FA. The dried films were highly flexible and conductive (see Fig. S4). The flexible hybrid film showed moderate mechanical strength with Young’s modulus of 0.22 GPa, comparable with polypropylene films (see SI).

We have investigated the time-dependent and thermal stabilities of the hybrid films in terms of electrical conductivity. As a result, conductivity of the hybrid film containing 75.5% HNTs slightly changed from initial value, 0.5 S/cm to 0.3 S/cm even after storage in atmosphere for two years. Similarly, conductivity of hybrid films treated with FA changes from 48 S/cm to 25 S/cm. On the other hand, the hybrid film after two-year storage also showed excellent thermal stability, i.e., initial value was 0.3 S/cm, and then 0.3 S/cm after thermal treatment at 50 °C for 30 min; 0.3 S/cm (80 °C); 0.3 S/cm (120 °C). The hybrid film treated with FA and EG also showed similarly good thermal stabilities, i.e., in the case of FA: 22 S/cm (initial); 26 S/cm (50 °C); 25 S/cm (80 °C); 25 S/cm (120 °C), and in the case of EG: 59 S/cm (initial); 59 S/cm (50 °C); 53 S/cm (80 °C); 50 S/cm (120 °C).

To verify the mechanism we observed pure HNTs and PEDOT/PSS-treated HNTs (immersed in PEDOT/PSS dispersion, filtered and dried) by TEM-EDX (Figs 4 and 5). The pure HNTs had clear and clean channels observed in the TEM image while the HNTs in the film had not any channels or not such clear and clean channels, moreover, narrower than the pure one (Fig. 4A,B). EDX spectrum showed the peak due to sulfur element and the intensity of the peak showed normal distribution across width of the NHTs in the case of PEDOT/PSS-treated one (see Figs 4C and 5). The observation revealed that the PEDOT/PSS entered into the channels of the HNTs in the hybrid films. In the UV-Vis-near IR spectra (see Fig. S7A) the PEDOT/PSS-HNTs hybrid films with EG (blue line) or FA (green line)-treatments showed strong tailing absorption in near IR regime, indicating PEDOT/PSS in the film is secondarily doped, which were resulted in significant enhancement of conductivity^8^,^9^,^10^,^11^,^12^, while pristine PEDOT/PSS film (black line) had no such tailing absorption. Interestingly the PEDOT/PSS hybrid film without any secondarily doping (red line) also showed such strong tailing absorption in the near IR regime. FT-IR spectra revealed that the HNTs and the PEDOT/PSS are physically mixed in the hybrid film (see Fig. S7B).

The enhancement of conductivity by addition of an insulating nanotube is considered to be a new phenomenon, which is different from that of addition of certain organic solvent such as DMSO and EG, and also is different from that of treatment of protonic acid such as sulfuric acid and formic acid. Here we propose new mechanism of the insulating nanotube-related enhancement of the conductivity based on our experimental data and observations. The typical mechanism is: 1) The aqueous PEDOT/PSS dispersion is absorbed into the nano-channels with diameter of ca. 30 nm of the HNTs due to the strong capillary effect; 2) The PEDOT/PSS colloidal particle with particle size of several tens nanometers well pack each other at the nano-channels into well-ordered structures of PEDOT/PSS particles, which show conductivity as higher as several order of magnitude than that of PEDOT/PSS particles in outside of the HNTs. Since the inner side of the HNTs is highly hydrophilic^20^, the nano-channels would show strong capillary effect which enables the PEDOT/PSS dispersion fully filling into the nano-channels of the HNTs. The filled PEDOT/PSS colloidal particles with a particle size of 16–40 nm well order and compactly pack each other due to restricted space of the nano-channels with a diameter of ca. 30 nm^7^,^20^. The well-ordered and packed domains would show higher conductivity than those of the PEDOT/PSS in outside of the HNTs. When the increase of the adding amount of the HNTs two trends of conductivity change would co-exist, i.e., 1) increase of conductivity due to increase of ordered domains, however, the increase stops when the fully filled state (see Fig. S7D); 2) and decrease of conductivity due to decrease of total conductive PEDOT/PSS component in the hybrid film (see SI). Combination of the two trends comprehensively show maximum value of conductivity when the content of HNTs increases (see Fig. S7D). The necessary amount of HNTs at the maximum conductivity can be estimated according to Eq. 1:


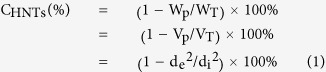


where C_HNTs_, W, V and d are necessary minimum amount of HNTs for full filling of PEDOT/PSS, amount in weight, amount in volume and diameter of the HNTs, respectively. The P, T, e and i denote PEDOT/PSS, total, external and inner, respectively. In Eq. 1 the density of both PEDOT/PSS solid and HNTs assume to be 1. Interestingly, when the d_e_ and d_i_ are 60 and 30 nm, respectively, the C_HNTs_ is 75%. It is agreement with the experimental data in Fig. 2. It was reported that conducting polymer can insert into nanocavity of nanostructures^23^,^24^.

Based on the proposed mechanism it is well explained for the changes of conductivity when the content of HNTs changed. The conductivity increased from 0.2 to 0.6 S/cm till maximum value when increasing amount of HNTs, which means enhancement of 3.7 times. However, the PEDOT/PSS occupies only 15% in the hybrid film, therefore, the enhancement should be 25 times or at least one order of magnitude. The enhancement indeed is significant but not high compared with solvent-addition and acid-treatment. It is well known that addition of EG or treatment of FA can improve conductivity of the PEDOT/PSS films by three orders of magnitude^6^,^9^. Therefore, when adding EG or treating with FA for the hybrid films the maximum point should be disappear and the conductivity straightforward decreases with increase in amount of HNTs, or with decrease in amount of PEDOT/PSS component. The results indicated that when the highly conductive PEDOT/PSS hybridized with the HNTs the second trend (see Fig. S7D) simply decides the total change of the conductivity of the hybrid films.

In conclusion we have for the first time found new phenomenon where conductivity of PEDOT/PSS film is significantly improved by mixing insulating HNTs. Based on experimental evidences we proposed a plausible mechanism of the phenomenon to explain the enhancement of the conductivity.

This work was supported by a “Key Project Seeds” fund in College of Chemistry and Molecular Engineering, Zhengzhou University, and by a “2014 Creative Talents in Science and Technology” fund in Henan province, China.

## Additional Information

**How to cite this article**: Yan, H. *et al.* PEDOT/PSS-Halloysite Nanotubes (HNTs) Hybrid Films: Insulating HNTs Enhance Conductivity of the PEDOT/PSS Films. *Sci. Rep.* **5**, 18641; doi: 10.1038/srep18641 (2015).

## Supplementary Material

Supplementary Information

## Figures and Tables

**Figure 1:**
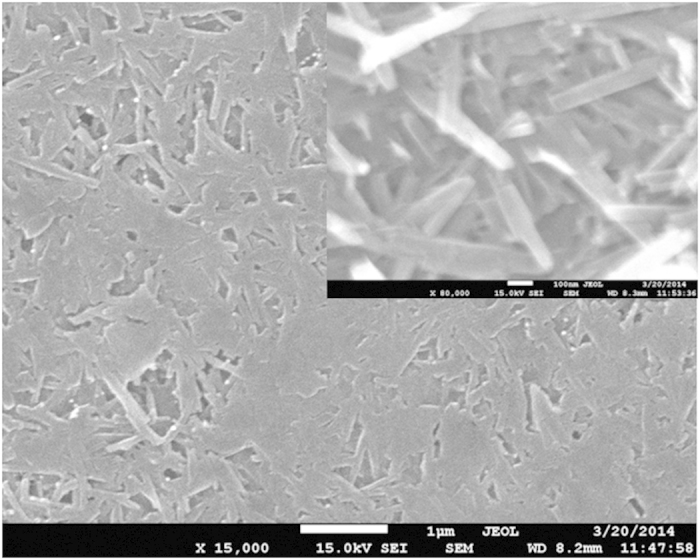
SEM image of the PEDOT/PSS-HNTs hybrid film prepared in the presence of EG. Scale bar: 1 μm but 100 nm in inset.

**Figure 2:**
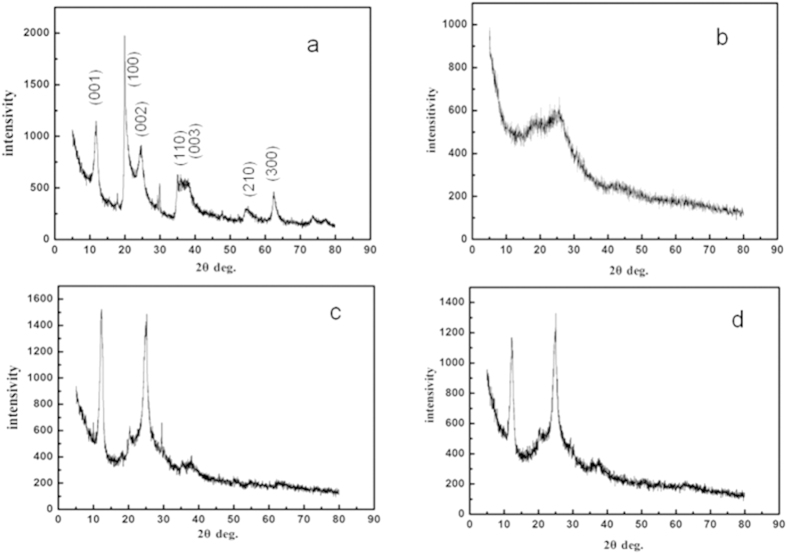
XRD patterns of the HNTs (**a**), PEDOT/PSS film (**b**), PEDOT/PSS-HNTs hybrid film (**c**) and PEDOT/PSS-HNTs film treated with FA (**d**).

**Figure 3:**
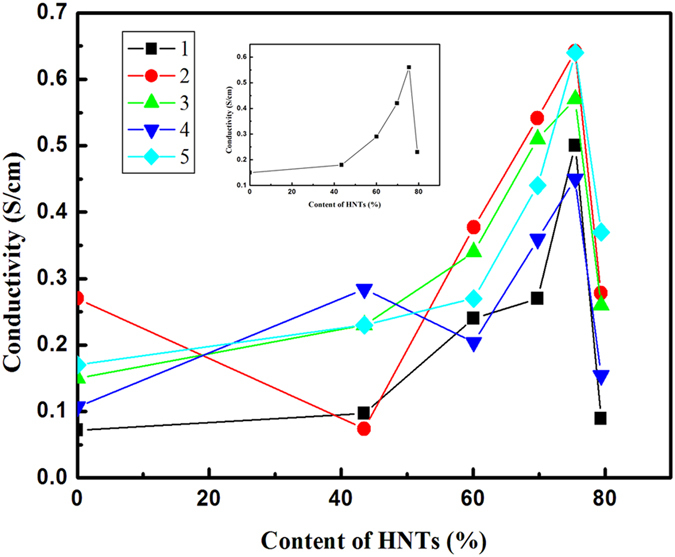
Conductivity of the PEDOT/PSS-HNTs hybrid films as content of the HNTs varied. The experiment was conducted repeatedly five times, and the averaged values were plotted in inset.

**Figure 4:**
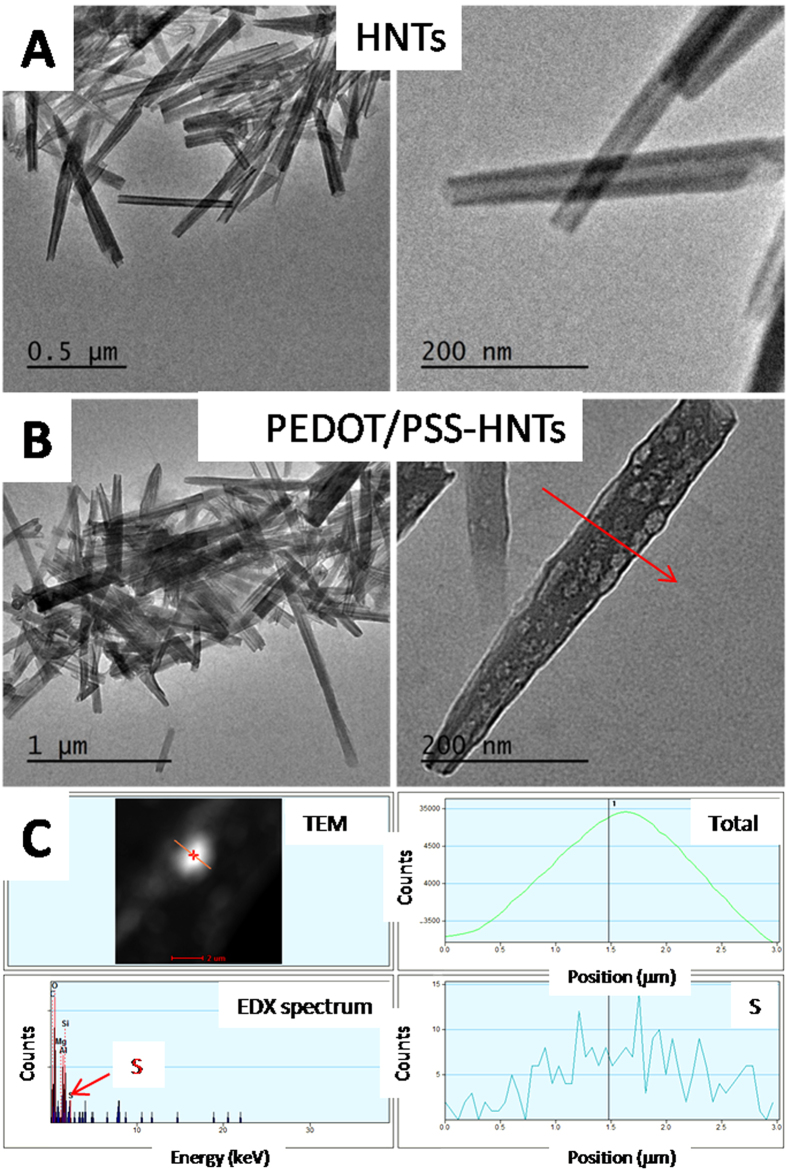
TEM images and EDX data of pure HNTs and PEDOT/PSS-HNTs. (**A**) TEM of pure HNTs; (**B**) TEM of PEDOT/PSS-HNTs (red arrow indicates scan direction of EDX); (**C**) EDX data of PEDOT/PSS-HNTs (left top: TEM; left bottom: EDX spectrum; right top: EDX intensity of total elements; right bottom: EDX intensity of sulfur element).

**Figure 5:**
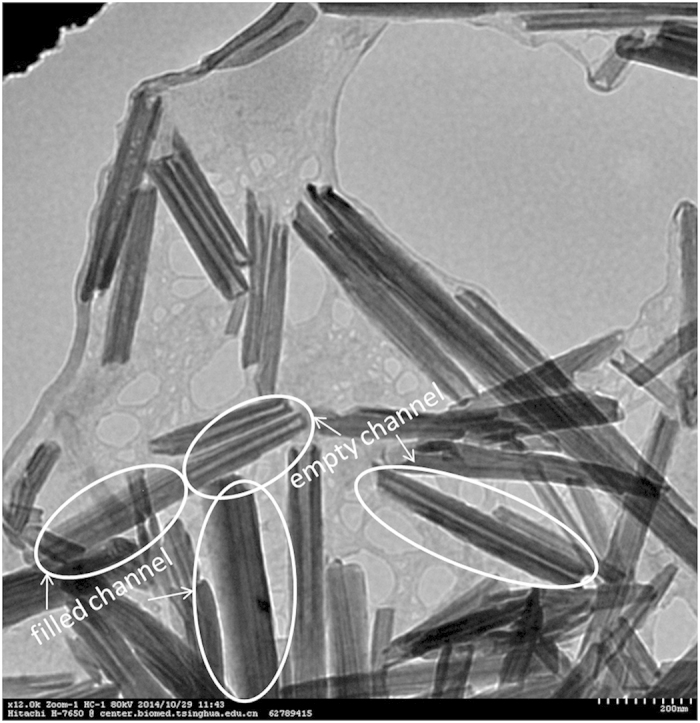
TEM image of the HNTs after immersed in PEDOT/PSS dispersion. Scale bar: 200 nm.
