# Supplementary material for: PEDOT/PSS-Halloysite Nanotubes (HNTs) Hybrid Films: Insulating HNTs Enhance Conductivity of the PEDOT/PSS Films
Source: Sci Rep. 2015 Dec 21;5:18641. doi: 10.1038/srep18641 (PMC4685446; doi:10.1038/srep18641)
Supplement: Supplementary Information [file srep18641-s1.pdf]

PEDOT/PSS-Halloysite Nanotubes (HNTs) Hybrid Films: Insulating HNTs Enhance Conductivity of the PEDOT/PSS Films

Hu Yan,[*,1] Ping Zhang,[1] Juan Li,[1] Xiao-Li Zhao,[1] Ke Zhang,[*,1] Bing Zhang[*,2]

[1]College of Chemistry and Molecular Engineering, and [2]School of Chemical Engineering and Energy, Zhengzhou University, Zhengzhou, Henan 450001, China

E-mail: yanhu@zzu.edu.cn; zhangke81@zzu.edu.cn; zhangbing1015@126.com

Experimental

1. Preparation of PEDOT/PSS-HNTs hybrid films:

Certain amount (10.7, 21.3, 32.1, 43.5, 54.7 mg) of HNTs was dispersed in certain amount (correspondingly 1011.5, 1039.0, 1040.3, 1044.2, 1039.2 mg) of the PEDOT/PSS dispersion (Clevios PH1000) and stirred for 24 h at room temperature. The HNTs-mixed PEDOT/PSS dispersion was drop-casted on poly(ethylene terephthalate) (PET) sheets (2 cm x 2 cm). The PEDOT/PSS-HNTs hybrid films on the PET sheets were first dried at 50℃ for 1-2 h, and then at 120℃ for 15 min.

2. Preparation of EG-treated PEDOT/PSS-HNTs hybrid films:

The ethylene glycol (EG)-treated films were prepared by using 7% EG-added PEDOT/PSS dispersion. Other procedures are the same with Section 1.

3. Preparation of FA-treated PEDOT/PSS-HNTs hybrid films:

The formic acid (FA)-treated films were prepared as follows: The PEDOT/PSS-HNTs hybrid films prepared in Section 1, were immersed in 88% FA for 10 min, peeled from the PET and then dried at 140℃ for 5 min.

4. Preparation of flexible PEDOT/PSS-HNTs hybrid films:

To improve the flexibility of the hybrid films certain amount (10.1, 35.1, 59.0, 81.6 mg) of polyethylene glycol (PEG), certain amount (correspondingly 44.1, 47.4, 46.7, 45.0 mg, which keeps 4% in the total mixture including the PEDOT/PSS dispersion) was added to the certain amount (correspondingly 1052.8, 1129.2, 1121.5, 1081.4 mg) of the PEDOT/PSS dispersion and the films were prepared in the same with Section 1. The films were further treated with the FA in the same with Section 3.

Measurements and Results

1. Measurements of electrical conductivity:

The sheet resistance of the films was measured by a standard four-probe method with a Resistivity Meter (Loresta GP Model MCP-T610). The thickness was measured with a micrometer. The electrical conductivity was calculated from the equation (1):

$$\sigma = \frac{1}{R_S \cdot d} \qquad (1)$$

Where σ, R_s and d are conductivity, sheet resistance and thickness of the film, respectively. For every sample the sheet resistance and thickness were at four different

positions and the values were averaged. We conducted the experiment where correlation between conductivity and HNTs-content of the hybrid films for five times.

For the EG-treated hybrid films the experiments and measurements were conducted in the same manner but only one time. The result was shown in Fig. S3C.

For the FA-treated hybrid films the experiments and measurements were conducted in the same manner. All results were shown in Fig. S1. We averaged the values of six experiments and newly presented in Fig. S3D.

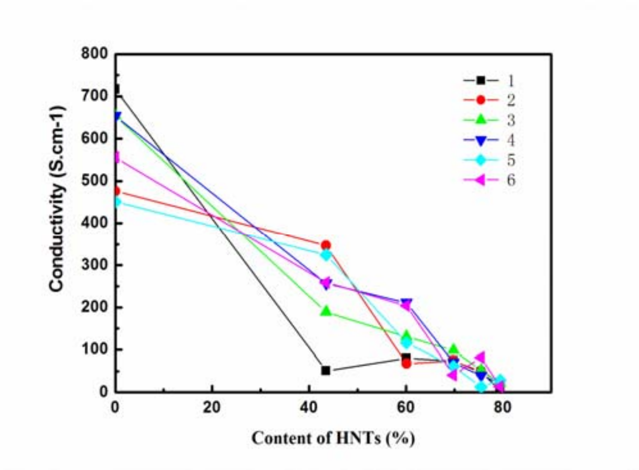

Figure S1. Conductivity of the FA-treated PEDOT/PSS-HNTs hybrid films as content of the HNTs varied. The experiment was conducted repeatedly six times.

For PEG-treated hybrid films the experiments and measurements were conducted in the same manner but only one time. The result was shown in Fig. S2.

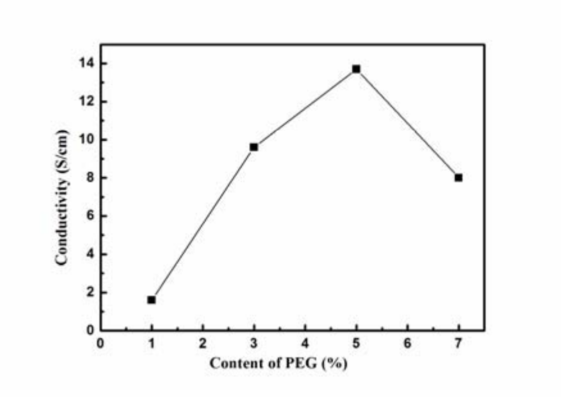

Figure S2. Conductivity of the PEG-treated PEDOT/PSS-HNTs hybrid films as content of the PEG in total dispersion varied but HNTs was kept in constant content against PEDOT/PSS (HNTs:PEDOT/PSS is 3:1 in weight) in the films. The composite film at the content of 5% PEG was investigated as the flexible hybrid film and further treated with FA.

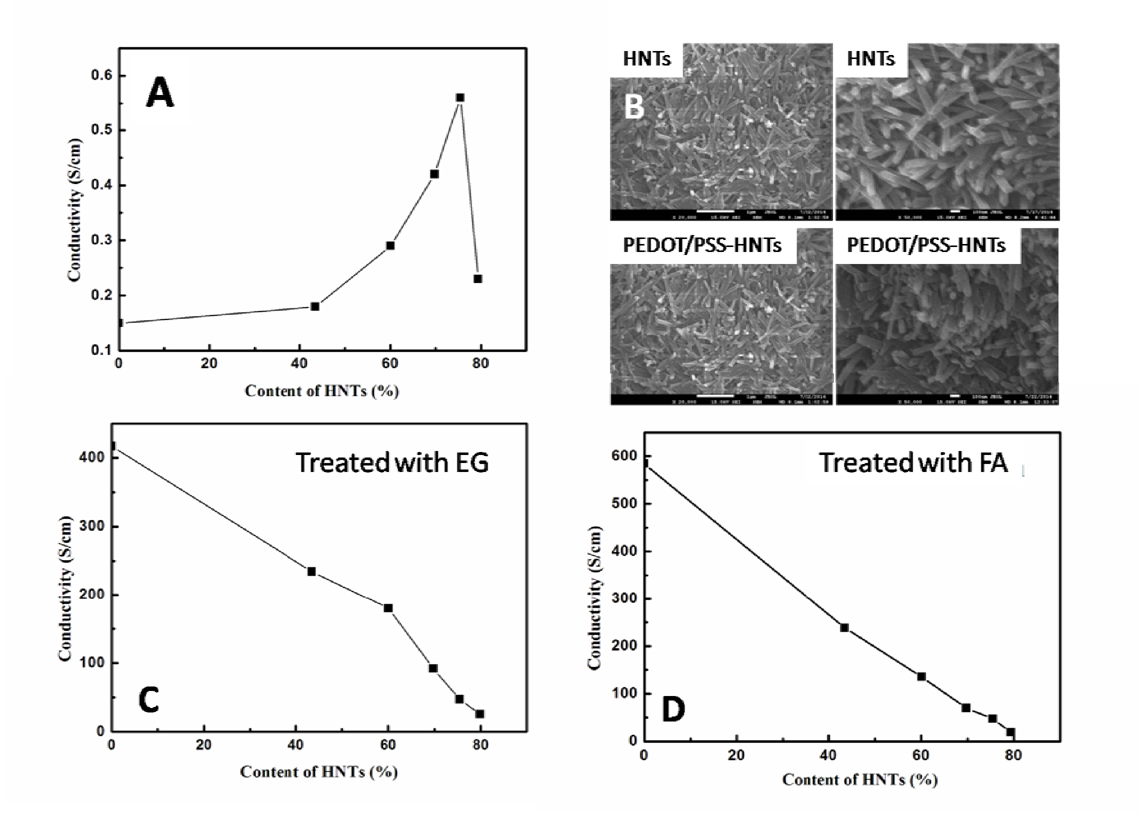

Figure S3. Conductivity of the PEDOT/PSS-HNTs hybrid films as content of the HNTs varied. A: before treatment; C: addition of EG during preparation of the films; D: after treatment with FA. B: SEM images of pure HNTs (top) and HNTs in the film (bottom). Scale bars in SEM: 1 μm (left) and 100 nm (right).

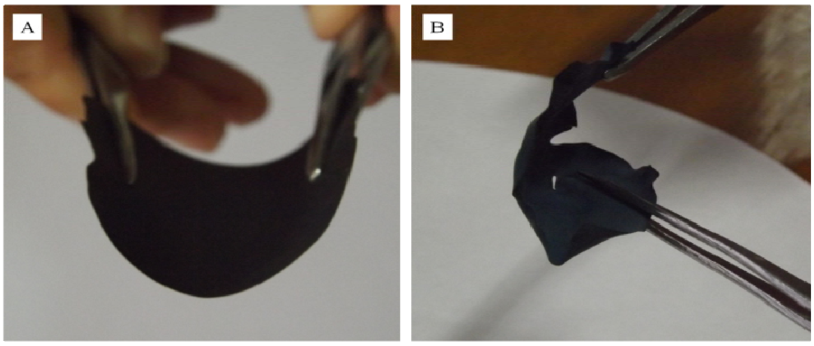

Figure S4. Photographs of the PEG-treated PEDOT/PSS-HNTs hybrid films. A: As prepared; B: Further treated with FA.

2. SEM measurements:

SEM images of the HNTs and the hybrid films were observed by a scanning electron microscope (SEM, JEM-2100cx). The specimen was sputtered with gold before the observations.

3. XRD Measurements:

XRD patterns were obtained using a X-ray powder diffractometer (Bruker AXS, D8 ADVANCE). Powdered sample of HNTs was studied by placing a thin layer in conventional cavity mounts while the films were adhered on the cavity mount. The samples were scanned from 0–80° in 2θ. The Cu anode X-ray was operated to give monochromatic Cu Kα X-rays (λ=1.5418 angstrom).

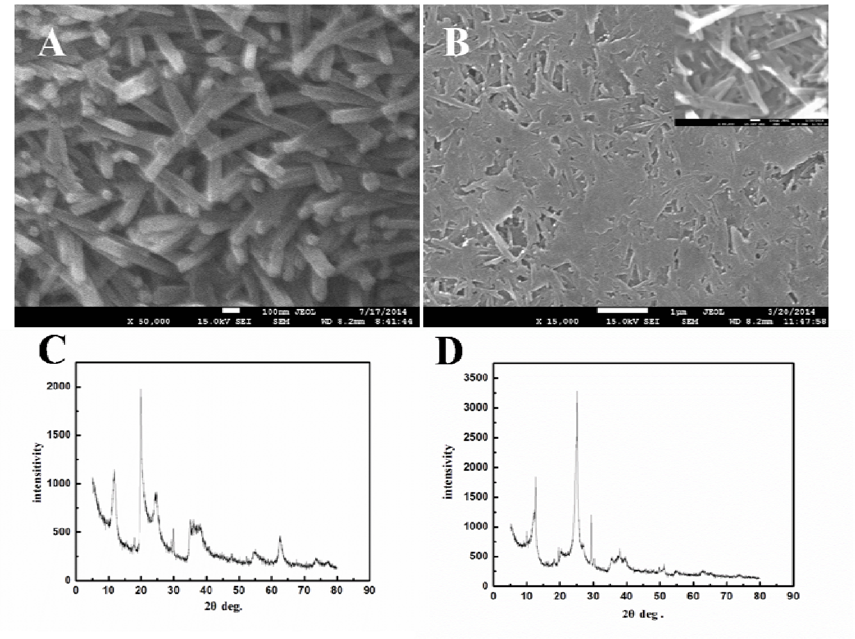

Figure S5. SEM images and XRD patterns of pure HNTs and PEDOT/PSS-HNTs hybrid film prepared in the presence of EG. A: SEM of HNTs; B: SEM of the hybrid film (scale bar in inset of B: 100 nm); C: XRD of pure HNTs; D: XRD of the hybrid film.

4. TEM-EDX Measurements:

TEM images and EDX spectra of the HNTs with or without PEDOT/PSS-treatment were measured by a transmission electron microscope (TEM, FEITECNAIG2). The specimen was prepared as follows: 43.5, mg of HNTs was dispersed in 1044.2 mg of the PEDOT/PSS dispersion (Clevios PH1000) and stirred for 24 h at room temperature. The HNTs were filtered and re-dispersed in the deionized water. The water dispersion of the HNTs was dropped on a copper grid and dried for the measurements. The pure HNTs were dispersed in deionized water. The dispersion containing HNTs was dropped on the copper grid and dried for the measurement.

5. BET Measurements:

BET measurements for surface area of pure HNTs and the hybrid films were conducted by a nitrogen adsorption technique using a NOVA 2000e Surface Area Analyser (Quantachrome), Moisture was removed from the samples prior to the surface area measurement (Fig S6)

6. UV-Vis-Near IR Measurements:

UV-Vis-near IR spectra of the hybrid films were measured by reflectance mode with a UV-Vis-near IR spectroscope (Agilent AU12320005) in the wavelength range of 300-2500 nm. The PET sheet and air were used as the reference for the PEDOT/PSS film on PET sheet and the hybrid films, respectively (Fig. S7).

7. FT-IR Measurements:

FT-IR spectra of the pure HNTs and the hybrid films were measured with a FT-IR spectrometer (Nicolet, NEXUS-470) by using pellet samples compressed with KBr. The spectra were shown in Fig. S7.

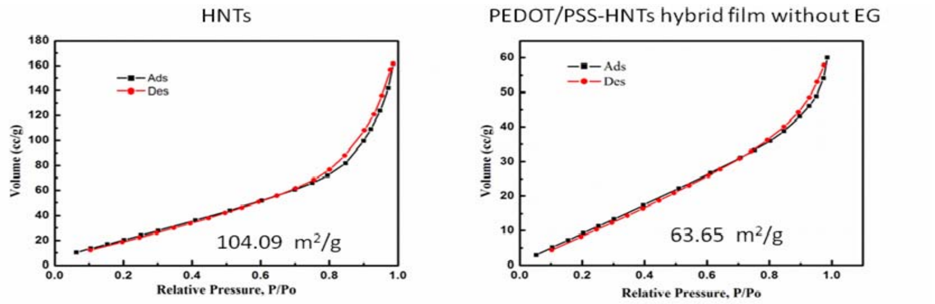

Figure S6. BET isothermal curves of pure HNTs (left) and PEDOT/PSS-HNTs hybrid film prepared without addition of EG(right).

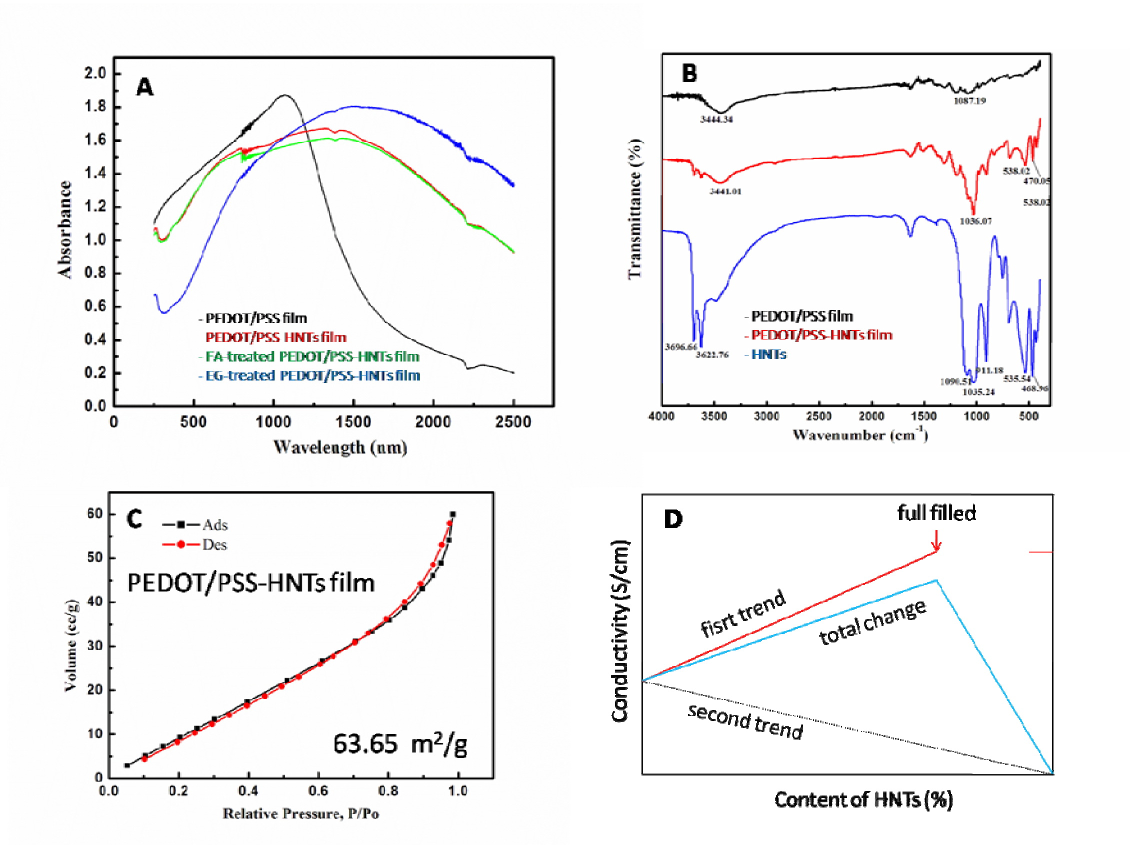

Figure S7. A: UV-Vis-near IR spectra of PEDOT/PSS based films; B: FT-IR spectra of pure HNTs, PEDOT/PSS film and the hybrid film; C: BET isothermal absorption/desorption curves of the hybrid film; D: Plausible correlations between the conductivity and the content of HNTs in the hybrid film.

8. Stress-strain curve measurements:

Stress-strain curves of the film samples were measured with a mechanical tester (UTM2203) in stretching rate of 5 mm/min. Rectangular shape (8-13 mm in length; 7-10 mm in width; 0.01-0.05 mm in thickness) of the films were used for the measurements. The results were shown in Fig. S6.

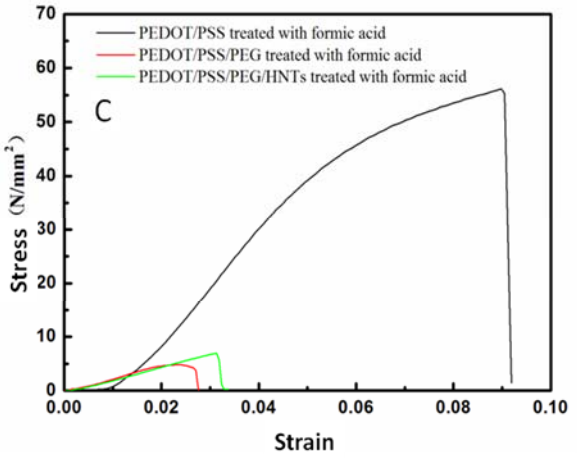

Figure S8. Stress-strain curves of the PEDOT/PSS-based films. Black line: PEDOT/PSS film treated with FA ; Red line: The PEDOT/PSS/PEG film treated with FA; Blue line: The PEDOT/PSS/PEG-HNTs hybrid film treated with FA.

**Sample**	**Young's modulus, E (GPa)**
PEDOT/PSS film treated with FA	0.78
PEDOT/PSS/PEG film treated with FA	0.24
PEDOT/PSS-HNTs film treated with FA	0.22
